# Spiral and Rotor Patterns Produced by Fairy Ring Fungi

**DOI:** 10.1371/journal.pone.0149254

**Published:** 2016-03-02

**Authors:** Nathaniel Karst, David Dralle, Sally Thompson

**Affiliations:** 1 Mathematics and Science Division, Babson College, Wellesley, MA, United States of America; 2 Department of Civil and Environmental Engineering, University of California, Berkeley, CA, United States of America; Universitat Pompeu Fabra, SPAIN

## Abstract

A broad class of soil fungi form the annular patterns known as ‘fairy rings’ and provide one of the only means to observe spatio-temporal dynamics of otherwise cryptic fungal growth processes in natural environments. We present observations of novel spiral and rotor patterns produced by fairy ring fungi and explain these behaviors mathematically by first showing that a well known model of fairy ring fungal growth and the Gray-Scott reaction-diffusion model are mathematically equivalent. We then use bifurcation analysis and numerical simulations to identify the conditions under which spiral waves and rotors can arise. We demonstrate that the region of dimensionless parameter space supporting these more complex dynamics is adjacent to that which produces the more familiar fairy rings, and identify experimental manipulations to test the transitions between these spatial modes. These same manipulations could also feasibly induce fungal colonies to transition from rotor/spiral formation to a set of richer, as yet unobserved, spatial patterns.

## Introduction

**Fig 1:**
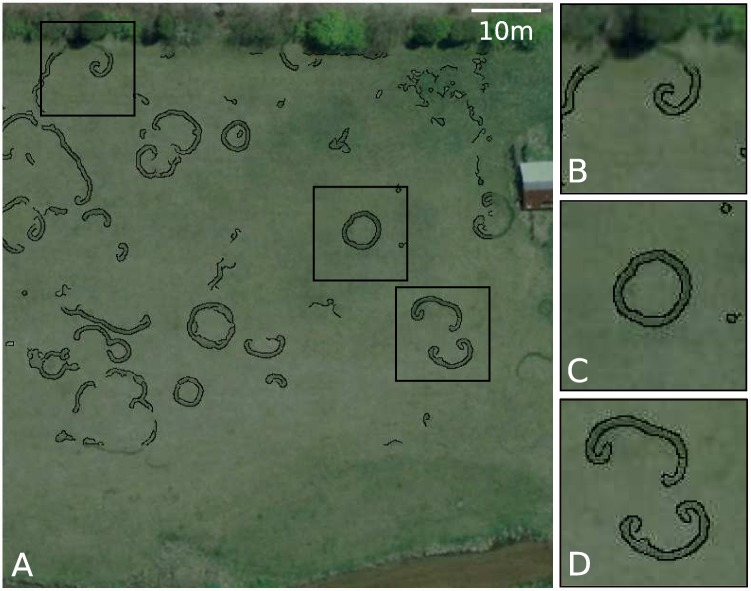
(A) Field near Boonsboro, MD, US (39.525678°, -77.768956°), in April 2008. Black traces identify edges between different grass greenness. We observe typical fairy rings (C) as well as isolated spirals (B), and rotors (D). Initial image capture from Google Earth. Imagery provided by the High Resolution Orthoimagery (HRO) program of the USGS.

Over 50 species of soil growing fungi, largely within phylum *Basidiomycota*, form colonies with an annular structure, known as a ‘fairy ring’, that propagates out from an initial point of innoculation [1–5]. The below-ground colony structure is revealed at the soil surface either directly by the presence of fruiting bodies, or indirectly through the effects of the fungal mycelia on surrounding vegetation—typically grasses. A range of negative effects (such as cyanide production, nutrient depletion, water repellency and pathogenic action of the fungi) lead to grass death within the fairy ring [6–9], while positive or hormonal effects (such as nutrient release, or the hormone-mimicking effects of fungal metabolites) promote leaf growth and local formation of a ring of luxuriant foliage in proximity to actively growing mycelia [10–12]. Fairy rings afford a valuable opportunity to observe the macroscopic spatio-temporal dynamics of soil fungi, because the association between vegetation condition and the presence of fungal mycelia is strong and simple (when compared, for instance, to the more complex task of inferring fungal pathogen dynamics from observations of plant disease [13]). While fairy rings and simple arc-shaped colonies have been widely reported for some time [1–3], high resolution aerial imagery made available in the last decade as seen in Fig 1 and in 19 other examples across the continental United States documented in the S1 File, show that colonies can also form spirals and rotors. To our knowledge, such structures have not been reported in the literature on fairy rings. The spirals formed on a wide variety of environmental conditions: soils ranged from highly drained gravels to poorly drained silts, and the climates from subtropical (Tennessee) to arid continental (Montana). There may be more commonalities in management practices: approximately half the sites are highly managed lawns (urban, golf courses or playing fields), and the other half mown hay fields and pastures. Regular mowing, fertilization and/or manure applications are likely to feature in the management of many of sites where spirals formed. At the same time, aerial imagery alone cannot tell us about potentially important differences among the sites, e.g. differences in fairy ring species, in neighboring turf species, and in soil conditions. Without a detailed physical inspection of all 20 sites, any interpretation linking these observations of spirals and rotors will remain speculative.

The growth processes of hyphae and mycelia within the soil are challenging to observe, and their kinetics, environmental dependencies, characterization (e.g. through appropriate rate constants) and up-scaling to the level of colony dynamics, remains an open field of investigation [14–16]. This cryptic behavior makes a direct investigation of the drivers of spiral pattern formation challenging: a challenge that we attempt to address here using mathematical modeling. In this context, we pose two goals for model application: (i) reproducing the qualitative pattern dynamics and (ii) generating testable experimental hypotheses which could be robustly interpreted, despite the considerable uncertainties likely associated with estimating specific model parameters or observing individual processes. Several models have been proposed to describe the bulk growth and mortality dynamics of soil fungal biomass, and their dependence on local concentrations of soil organic matter and bio-available nutrients, collectively known as “substrate” in the mycology literature, but which we refer to here as “resources” to more clearly indicate their role [4, 17–21]. When these dynamics propagate through space (enabled by diffusion of solutes and growth of fungal hyphae through the soil [22]), the resulting colony forms an annulus of high fungal biomass, propagating outwards away from areas where resources have been consumed and into un-colonized regions of the soil [1, 3, 23]. Although formation of temporally stable spatial patterns, chaotic oscillations and traveling waves has been observed in numerical simulations of biomass-resources equations (see [4, 17, 18]), the main goal of modeling studies, to date, has been to reproduce the widely-observed fairy ring morphology.

In this study, we present a prototypical fungal biomass-resources reaction-diffusion model and demonstrate that it is isomorphic to the well-studied Gray-Scott model [24], which is known to support a number of pattern-forming and dynamical regimes across its parameter space [25]. Bifurcation and numerical analysis are then used to explore the region of dimensionless parameter space that supports these spatial dynamics. We show that this region in parameter space overlaps with and lies adjacent to the region supporting formation of annular fairy rings. We identify the stability boundaries for fairy rings, ‘fairy spirals’, and beyond, finding neighboring regions of parameter space that support regular spatial pattern formation. Finally, we propose and numerically evaluate an experimental test for the hypothesis that fairy rings should evolve into spirals following a simple physical perturbation, without requiring manipulation of environmental or biological control parameters.

## Methods

In this section we outline numerical methods to explore the formation of spirals and rotors in fairy ring fungi. We begin by showing the equivalence of a simple model for fairy ring kinetics and the classic Gray-Scott reaction-diffusion model. With this approach in mind, we present the simulations used to assess initiation of spirals, the simulations used to explore parameter space, and the classification procedure adopted to generate our main results.

### Equivalence of fungal biomass model with Gray-Scott Equation

There have been many attempts at modeling fungal kinetics, both at the scale of individual hyphae [26–28] and at length scales in which the fungal colony is better approximated as a spatially distributed continuum of biomass [17, 29, 30]. In the absence of rigorous experimental validation of these models or a consensus among the mycological community as to which best captures fairy ring kinetics, we choose to adopt the most parsimonious of these models due to Davidson et al. [17]: a reaction-diffusion model describes the space-time evolution of fungal biomass density *b*(**x**, *t*) [mg m^−2^] and resource concentration *r*(**x**, *t*) [mg m^−2^], (where the Cartesian vector **x** represents the spatial coordinates), according to the following equations:

$$\begin{array}{cc} r_{t} & =D_{r}\Delta r-c_{1}rb^{2}+g\left(r_{max}-r\right) \end{array}$$ (1)

$$\begin{array}{cc} b_{t} & =D_{b}\Delta b+c_{2}rb^{2}-mb. \end{array}$$ (2)

Here, *m* [day^−1^] represents the mortality rate for the fungal biomass, and *g* [day^−1^] represents the rate at which resources are replenished towards their saturated value *r*_*max*_ [mg m^−2^]. The Laplacian is represented by *Δ*. Parameters *D*_*r*_ and *D*_*b*_ [m^2^ day^−1^] are the diffusion coefficients for resources and biomass, respectively. Fungal uptake of resources is captured in the term *rb*^2^, which adopts a autocatalytic (density-dependent) rate of resource exploitation. At the hyphal level, the biological justification for this density-dependence lies in the facilitation of resource uptake by the production of enzymes and other metabolites—whose production rates are themselves dependent on resource uptake [17]. In the continuum description of fungal biomass adopted here, density dependence relates to the increased efficiency with which a locally dense hyphal network can locate and translocate spatially discrete soil resources to the entire mycelial body [31, 32], increasing the efficiency of resource uptake with increasing biomass density. The parameter *c*_1_ [mg^−2^ m^4^ day^−1^] represents the rate at which available resources are mobilized and absorbed by the fungal biomass, and *c*_2_ [mg^−2^ m^4^ day^−1^] accounts for the efficiency and stoichiomtery of converting utilized resources into fungal biomass. These equations are a simplified representation of carbon translocation and use by mycelia. More complete and complicated representations of carbon fluxes account for both external resources and those that have been internalized by the fungal colony [27, 29, 33]. Accurately modeling these resource stores is further complicated by the fact that carbon translocation is a function of both diffusive and active transport [34, 35]. The simplifications used by Davidson et al. are analytically justified when quasi-steady state conditions can be applied to the internal and external carbon pools, a situation that arises when biomass growth is carbon-limited [36]. One could also consider more carefully modeling the kinetics of hyphal growth. While new branches do occasionally split off along the hyphal wall, individual hyphae tend to extend in a straight line, and so there has been some work to include a convective term to account for preferential forward growth [33]. The simplifications in the Davidson model assume that the effects of preferential growth average out locally, and so diffusion captures the overall trend.

Mycelia will globally respond to heterogeneous resources by taking up resources beyond local need in nutrient-rich areas and subsequently distributing the excess to nutrient-poor areas [34, 35], and models have been proposed that capture this phenomenon [29, 33]. While such behavior is likely to be quite important for small and medium scale fungal colonies in which nutrients can be conveyed from any part of the colony to any other part in a short amount of time, this internal redistribution will likely play a much smaller role in very large scale fungal colonies like fairy rings. Hence, the assumption in the Davdison model that resource draw-down is a function only of *local* biomass density seems well justified in this large colony limit.

To nondimensionalize Eqs 1 and 2, we first define the following (dimensionless) variables:

$$\begin{array}{c} \hat{r}=\frac{r}{r_{max}},\hat{b}=\frac{b}{r_{max}},\hat{t}=r_{max}^{2}c_{1}t,\hat{x}=\frac{r_{max}\sqrt{c_{1}}}{\sqrt{D_{b}}}x. \end{array}$$ (3)

Note that this choice of dimensionless variables is different than that of Davidson et al. [17]. These new variables induce the dimensionless parameters

$$\begin{array}{c} \hat{D}=\frac{D_{r}}{D_{b}},\;\hat{g}=\frac{g}{r_{max}^{2}c_{1}},\;\hat{m}=\frac{m}{r_{max}^{2}c_{1}},\;\hat{c}=\frac{c_{2}}{c_{1}}. \end{array}$$ (4)

We assume that for a given fungal species growing in a fixed environmental location, both the resource saturation level *r*_*max*_ and resource uptake rate *c*_1_ can be treated as constants. This implies that the dimensionless and dimensioned resource replenishment rates $\hat{g}$ and *g* are equivalent; a similar equivalence applies to the dimensionless and dimensioned fungal mortality rates $\hat{m}$ and *m*. With these definitions, we write a fully dimensionless description of the system:

$$\begin{array}{cc} {\hat{r}}_{t} & =\hat{D}\Delta \hat{r}-\hat{r}{\hat{b}}^{2}+\hat{g}\left(1-\hat{r}\right) \end{array}$$ (5)

$$\begin{array}{cc} {\hat{b}}_{t} & =\Delta \hat{b}+\hat{c}\hat{r}{\hat{b}}^{2}-\hat{m}\hat{b}. \end{array}$$ (6)

This dimensionless formulation is used throughout the remainder of the paper, dropping the “^” notation. Defining three new parameters and renaming state variables to avoid confusion,

$$\begin{array}{c} U↔r,\;V↔b,\;F↔g,\;k↔m-g,\;C↔c, \end{array}$$ (7)

we transform Eqs 5 and 6 into the canonical Gray-Scott equations:

$$\begin{array}{cc} U_{t} & =D\Delta U-UV^{2}+F\left(1-U\right) \end{array}$$ (8)

$$\begin{array}{cc} V_{t} & =\Delta V+CUV^{2}-\left(F+k\right)V. \end{array}$$ (9)

### Parameterization

The simulations considered variations in the dimensionless mortality (*m*) and dimensionless susbtrate replenishment (*g*) rates, as these form potential control parameters amenable to experimental manipulation. The diffusion rates were assumed to be primarily controlled by soil properties, which influence both the pattern and rate of hyphal expansion, and the diffusivity of solutes within the soil (see, e.g., [16]). We assumed that the diffusivity of resources is comparable to the diffusivity of water in soil (a value that spans many orders of magnitude, but can readily be as high as 10^−5^ m^2^day^−1^ for wet soils [37]), and that it was therefore reasonable to assume that the diffusion ratio for resource spread to hyphal spread was greater than 1. We used *D* = *D*_*r*_/*D*_*b*_ = 2 throughout, following previous analyses of both the Gray-Scott model [25, 38] and fairy ring fungi [4] which adopted this parameterization. We note that while this parameterization is fairly unconstrained, it also represents a conservative choice, in that larger values of *D* tend to enlarge the pattern-forming regimes within parameter space [4, 17, 20, 38]. There has been some interesting analytic work on tying the wave speed of the dimensionless Gray-Scott system to the system’s parameters, including the diffusivity ratio *D*[39, 40]. In some sense, such an approach would be ideal, in that we could use the well documented fairy ring wave speed to back out the less well known diffusivity ratio. Unfortunately, our scaling constants for both space and time contain unknown (and difficult to measure) quantities (e.g., *r*_*max*_ and *D* itself), and so transforming the well known dimensioned wave speed into its dimensionless equivalent introduces more problems than it solves. There has been to our knowledge only one set of studies which attempts to directly measure the diffusivity of soil fungi. Boswell et al. [33, 41] observed hyphal tip velocity of *R. solani* and translated these measurements into a fungal diffusivity, but their framing depends on the density of internalized resources, and so is not immediately applicable here.

## Numerical Simulations

**Fig 2:**
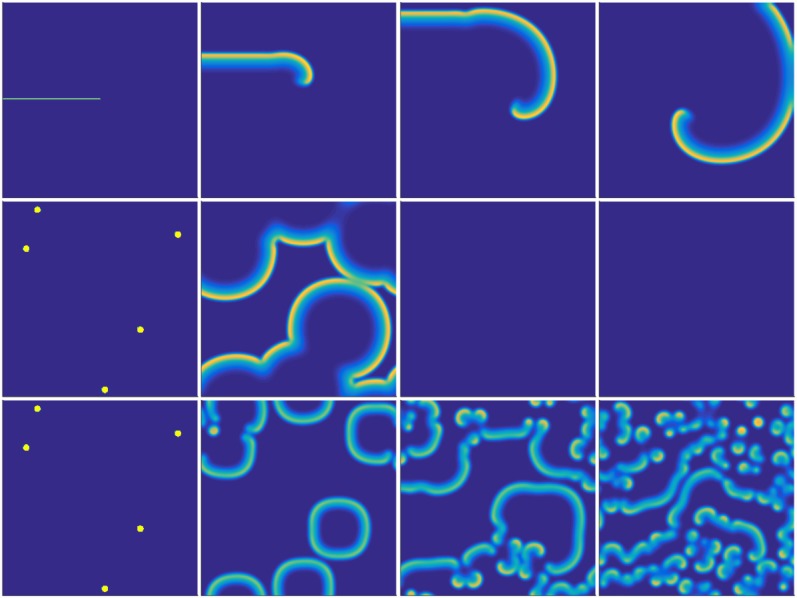
Evolution of biomass concentrations in typical simulations. Top row and middle row have identical dimensionless parameter values (*m* = 0.5, *g* = 0.01), showing that the colony can form both spirals and rings under appropriate initial conditions. Notice that the rings in the middle row mutual annihilate on contact. Middle row and bottom row have identical initial conditions but different dimensionless parameters (top row: *m* = 0.5, *g* = 0.01; bottom row: *m* = 0.65, *g* = 0.15). In the bottom row, byproducts of front collisions evolve into rotors. Snapshots were taken every 500 dimensionless time units beginning at *t* = 0.

Simulations were run with two different initial conditions: a line source and multiple point sources of fungal biomass. Prototypical simulation results can be seen in Fig 2. The line source was considered because spirals are known to form around the free end of linear fronts in similar systems [42, 43]. The multiple-point initial conditions create a situation where the only mechanism for spiral formation lies in the interaction of propagating fronts—these initial conditions much more closely resemble those we would expect to find in nature. The details of the numerical methods used to approximate both classes of simulation are found in S1 Text.

## Results

### Bifurcation analysis

**Fig 3:**
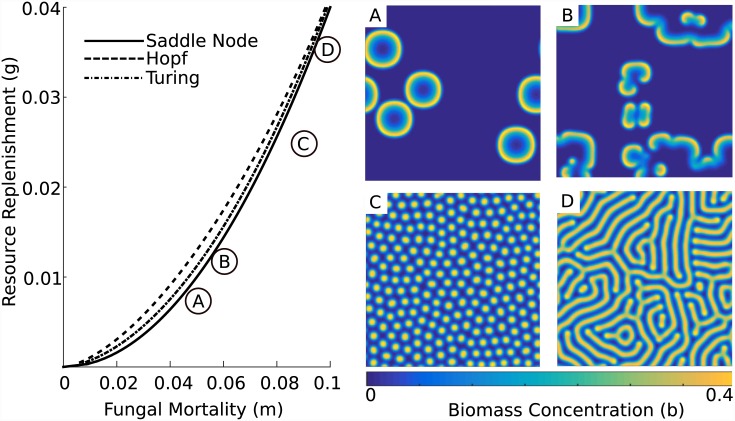
Examples of biomass evolution for several combinations of dimensionless parameters *m* and *g*. The system displays a wide range of behaviors, even in the region in which there is a single homogeneous equilibrium, including (A) mutually annihilating traveling waves akin to fairy rings, (B) complex spatiotemporal patterns that include spirals and rotors, (C) spots of high biomass concentration separated by barren regions, and (D) strands of high biomass concentration. Snapshots taken at (dimensionless) time *t* = 200, *t* = 1,500, *t* = 10,000 and *t* = 10,000 with parameters (*m*, *g*) = (0.0475, 0.0075), (*m*, *g*) = (0.058, 0.012), (*m*, *g*) = (0.085, 0.025), and (*m*, *g*) = (0.095, 0.035), respectively, and *D* = 2 and *c* = 1.

The Gray-Scott equations are known to support a suite of dynamical behaviors, including Turing patterns, spiral wave formation, phase turbulence, and spiral chaos [24, 25, 38]. The isomorphism between the fairy ring dynamical model and the Gray Scott equations therefore suggests the potential for fungal biomass-resource dynamics to generate richer behavior than annular fairy rings. A detailed review of known Gray-Scott dynamics is provided by Mazin et al. [38]. The most relevant features of the model for analyzing fairy ring behavior are summarized here, with reference to the bifurcation diagram shown in Fig 3.

In the absence of diffusion, and at low ratios of resource replenishment to fungal mortality, only one steady state exists: zero fungal biomass. At higher replenishment rates, a saddle node bifurcation occurs (solid curve in Fig 3), resulting in the creation of two new steady states, each with non-zero fungal biomass. One such state is always a (linearly unstable) saddle node, while the other undergoes a *Hopf bifurcation* (dashed curve in Fig 3) as the replenishment rate continues to increase, implying the existence of oscillatory dynamics around the steady state. When diffusion affects the evolution of the system, the same equilibrium undergoes a *Turing bifurcation* (dot-dashed curve in Fig 3), leading to the formation of spatially regular and temporally stable patterns. The region just above the saddle node bifurcation curve supports a tremendous array of dynamics through the interactions of the Hopf, Turing and saddle node bifurcations [25, 38, 44, 45]. However, since there is no experimental evidence to support the existence of nonzero biomass steady states in the field, our focus is on the less-studied region of parameter space, lying below the saddle node bifurcation. In this regime there is insufficient resource replenishment to support persistent fungal biomass, and in the absence of diffusion, the zero biomass state is the unique homogeneous equilibrium. The uniqueness of the zero-biomass equilibrium suggests that in the presence of diffusion, relatively high rates of resource transport are needed to support fungal growth (low transport rates approximate the diffusion-less case, so fungal biomass will still decay to the zero steady state). Rapid rates of transport require large spatial gradients in biomass and resources: in practice, this places an upper bound on the width of a fungal front, and implies that persistent biomass structures will be characterized by sharp biomass edges [25].

A wide range of behaviors arise below the saddle node bifurcation in the model system. It is primarily here that typical fairy rings form: an injection of biomass generates annular traveling waves behind which the system returns to its ground state (see Fig 3A). More complex patterns are also possible. Intersecting fronts of propagating fungal biomass may generate rotors or spirals (see Fig 3B). Pattern formation (e.g. spots and labyrinthine patterns) is also possible here—even in the absence of a Turing bifurcation—as a consequence of the excitable media dynamics prevailing in this region in parameter space (see Fig 3 panels C and D) [25].

### Rotor and spiral formation

**Fig 4:**
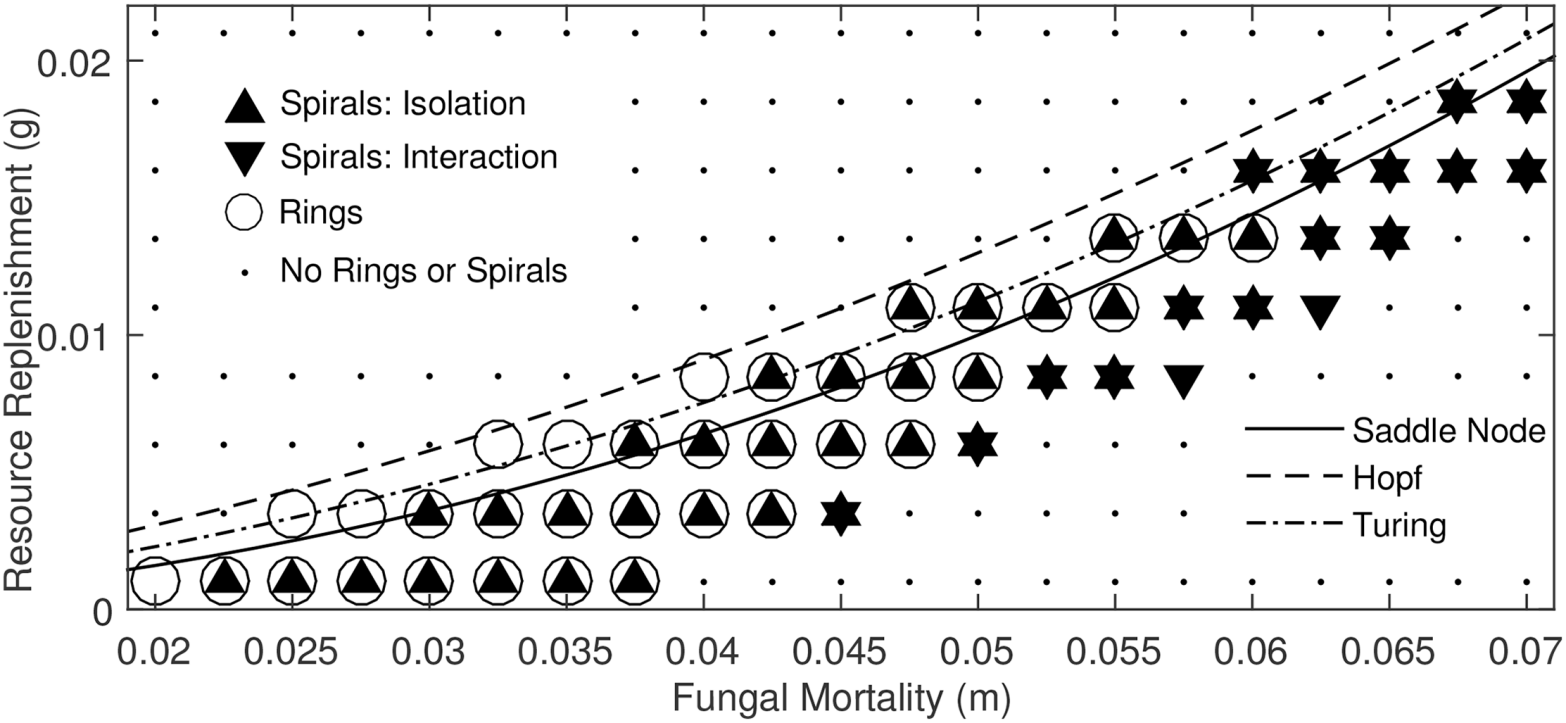
Results of numerical search for spiral formation across dimensionless parameter space. Upward-facing triangles indicate parameters supporting spiral formation only from an isolated linear front, downward-facing triangles represent parameters supporting spiral formation only through interaction of multiple fronts. Black circular outlines represent points at which inocula decayed to the ground state via an annular traveling wave, equivalent to typical fairy ring behavior. Small black dots represent parameters that were found to support neither rings nor spirals. Note that some (*m*, *g*) pairs can support multiple types of behavior due to differing initial conditions. Here, *D* = 2 and *c* = 1.

Formation of typical fairy rings was tested by introducing individual point sources of biomass. Numerical exploration showed that spirals and rotors can come about via two distinct dynamical pathways: through rotation induced at the free end of a linear fungal colony (in good agreement with other work on excitable media dynamics [42, 43]) and through the interaction of multiple fairy ring fronts. While fairy rings are often assumed to mutually annihilate when they collide, in a certain region of parameter space collisions occasionally generate free edges that act as a center of rotation for subsequent spiral/rotor formation. Having found these mechanisms for pattern generation, we undertook a numerical exploration of the model’s dimensionless parameter space to define the stability boundaries for spiral, rotor, and ring dynamics. All initiation mechanisms (point, linear and multiple points) were tested at each parameter combination. This allowed us to determine if a given region in parameter space supported the generation of fairy rings, isolated spirals, spirals that formed from the intersection of fronts, or a combination of these mechanisms. Results are shown in Fig 4.

Fig 4 shows that the regions of dimensionless parameter space which support typical fairy rings and spiral formation from isolated linear fronts almost perfectly coincide. The region of parameter space in which rotors/spirals form through the collision of fronts lies adjacent to the region in which fairy rings form, generally under higher fungal mortality and resource replenishment rates than the isolated fronts.

## Discussion

The dimensionless parameter spaces supporting fairy ring and fairy spiral formation from isolated linear colonies almost perfectly overlap, yet fairy rings are much more commonly reported than fairy spirals. In documenting the 20 sites featuring fairy spirals as detailed in S1 File, for instance, several hundred sites displaying only the typical ring morphology were found. Two factors are likely responsible for the relatively infrequent observation of spirals.

**Fig 5:**
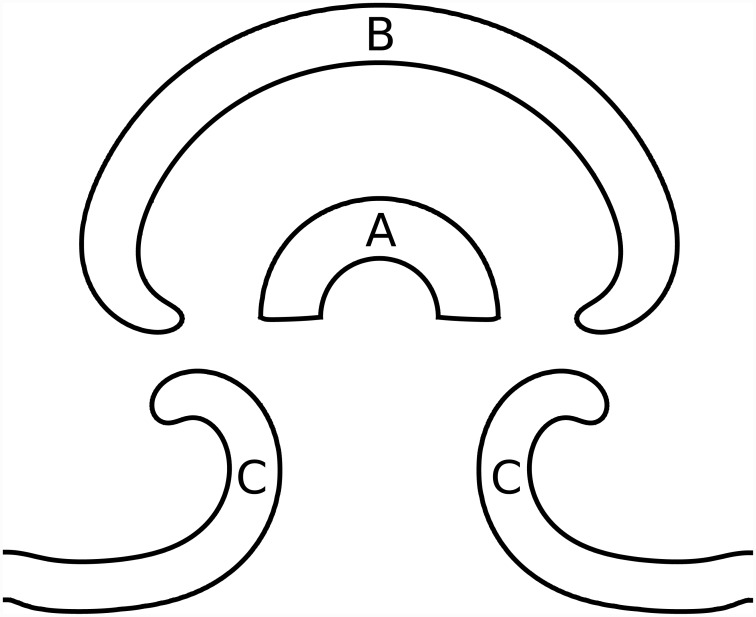
Simulation of one possible experimental mechanism for inducing fairy rings to form rotors. Edges represent contours at which biomass concentration *b* is equal to 0.1. (A) Half of an existing colony is replaced by a zero fungal biomass and low resource medium at (dimensionless) time *t* = 200. (B) The free edges of the colony begin to curl at *t* = 600. (C) The colony develops into a self-sustaining rotor at *t* = 1; 300; rotor tips were originally coincident with the curled tips in (B) and were translated down in the plane for clarity.

**Fig 6:**
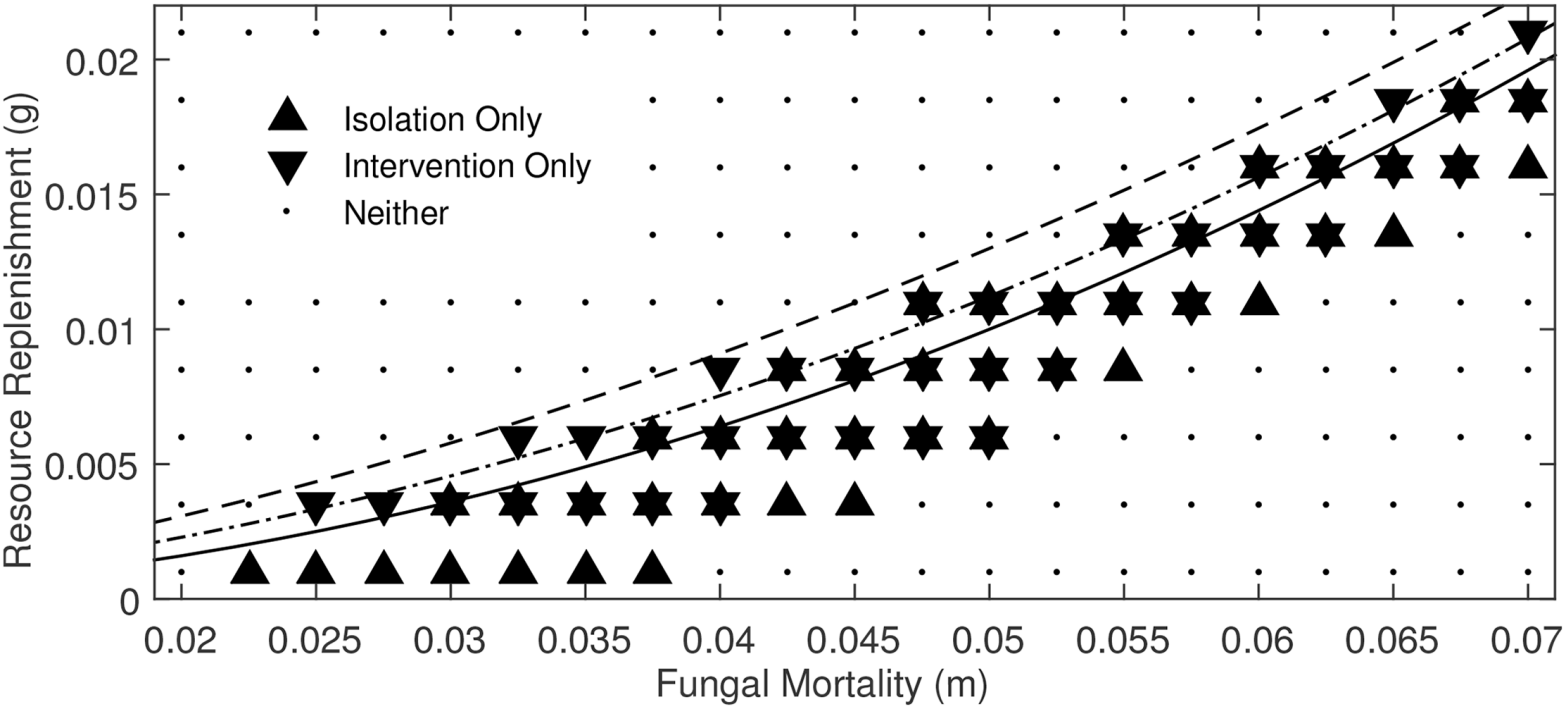
A comparison of areas of dimensionless parameter space supporting spiral formation through development of an isolated linear front and development via intervention in which one half of an existing ring is replaced with a zero biomass, low substrate medium. The two methods are nearly equivalent, with the most notable difference being that the lowest replenishment rates only support the spiral development of linear fronts. This is likely a numerical rather than practical effect; substrate replenishment is so slow that the remnants of a sliced ring grow off the simulation domain before the colony’s passage can be supported in the newly introduced medium.

Firstly, the initial conditions required to form fairy spirals in the most widely encountered biological and environmental conditions (i.e. those that support fairy rings), probably occur infrequently. The *Basidiomycota* disperse as spores, and colonial growth is initiated from an individual spore—i.e. a point biomass source. Thus, the dispersal ecology of fairy ring fungi is not conducive to generating the linear biomass fronts needed to induce spiral formation under ‘normal’ conditions. We note, however, that despite the infrequent observation of spirals, incomplete fairy rings (fairy arcs), with free edges are commonly seen and widely reported [3, 7]. It is unclear whether the widespread observation of free-edges that do not rotate poses a fundamental challenge to the predictions of the Davidson model, or whether observed arcs arose from processes that would not be expected to create spirals (e.g. intersecting fronts in a low *m* and low *g* parameter regime). To resolve this question, we propose a manipulative experiment that attempts to induce an existing fairy ring to form a spiral: remove a portion of the ring and replace the displaced soil with a low-resource medium (e.g. sand)—thus creating two free edges that can act as centers of rotation for self-sustaining rotors. A model realization of such a manipulation is shown in Fig 5. The re-analysis seen in in Fig 6 of the system’s parameter space indicates that this mechanism results in rotor formation for almost all parameter combinations that support ‘typical’ fairy rings. This manipulation therefore offers a robust test of the feasibility of this spiral forming mechanism and its co-incidence with fairy-ring formation in parameter space, despite the inherent uncertainties associated with model parameterization.

Secondly, it seems plausible that the biological and environmental conditions that allow intersecting fairy rings to create spiral waves may be unusual. Fig 4 suggests that to initiate spiral patterns from colliding fronts requires that mortality rates and resource replenishment rates are elevated relative to ring-forming conditions. Various feedback mechanisms in the environment may mitigate against reaching such conditions: for instance, fertilization experiments suggest that fungi do not necessarily increase growth rates when carbon is added to soils, because this carbon is more rapidly assimilated by bacteria [14]. However, there are plausible situations that could simultaneously stress fungi and provide rapid resource replenishment, such as a combination of soil compaction (which may stress fungi [16]) and regular mowing (which could increase resource delivery to the soil surface). These conditions could be consistent with frequent mowing and management of turf or hay production, and thus with the observations in Fig 1 and the sites in S1 File. Even under ideal conditions for spiral formation through front interaction, the environment must be stable over a period of decades to provide sufficient time for spirals and rotors to develop. Given the long timescales and multiple front interactions needed to generate spirals and rotors through this mechanism, we suggest that experimental manipulations intended to elucidate this pathway of spiral formation may be too challenging for field studies. An alternative approach could include a global ‘sweep’ of available aerial imagery to identify spiral patterns in fairy rings, and to explore backwards in time to identify the history of spiral formation. We note that this approach also poses significant challenges: the aerial imagery must be taken at a time of year when fairy rings are visible, requires high spatial resolution, and needs to span a period of 10–20 years. Indeed, we have (so far) been unable to find historical photographs showing the origin of any of the fairy spirals presented in S1 File.

Experimentally determining the source of the large disparity in frequency of occurrence of rings and spirals presents its own set of challenges. Indeed, even producing a single spiral in a controlled setting might require operating over rather long timescales. Fairy ring fungi exhibit radial growth rates on the order of 10 cm per year [23], even when grown under ideal laboratory conditions [9]. Our numerical simulations indicate that fairy ring’s diameter must be large compared to the width of the fungal front in order for spirals to form either through interaction of multiple fronts or through the cutting procedure described above. If the diameter is small relative to the width of the fungal front, the colony more closely resembles a disk than an annulus, and neither interactions nor interventions produce the isolated free tips necessary to induce spiral rotation. Assuming the fungal front of a fairy ring is roughly 1 meter wide [3], we should expect that only fairy rings with diameters on the order of several meters or more should be able to form fairy spirals. Cultivating a fairy ring of this size would take years, and simulations indicate that allowing spirals and rotors to fully develop after front interaction or intervention would take several times longer—on the order of a decade. Hence, even in the case of the experimental manipulation presented in Fig 5 which begins with a fully formed fairy ring, the relevant timescales may prove too long for straightforward empirical verification of our predictions. These difficulties cannot be circumvented by initially inoculating a linear front as seen in Fig 2; assuming a growth rate of 50 cm per year, a point 1 m from the center of rotation would take roughly 12 years to make a complete rotation.

Given the time, space, and data constraints on experimental tests of the transitions predicted in Fig 4, detailed experimental explorations of parameter space are likely to need small-scale, rapidly growing analogues to fairy rings that could be manipulated in laboratory settings (for instance, by altering carbon fertilization rates, *g*). If such analogues can be found, a particularly attractive experiment would test the existence of the patterned regimes identified in Fig 3C and 3D, which require high resource replenishment (*g*), high mortality (*m*), and opportunities for multiple front interactions before expanding rings adopt stable, regular spatial patterns. Such experiments would be compelling for two reasons. First, they would help more clearly demarcate the regions of parameter space in which the Davidson model is a useful approximation of fungal kinetics. Second, the observation of any such patterns in the laboratory would motivate the search for as-yet-unobserved behaviors in the wild.

## Conclusion

Our results suggest that fairy rings can adopt more complex spatial forms than simple annuli, and that they appear to do so in a manner that is consistent with the predictions of the simple Davidson model. The experiments and imagery exploration proposed here would provide further tests of this consistency, relying on qualitative dynamics that should be robust to the specific environmental conditions under which the tests are performed. Further advances in understanding fairy ring dynamics, drawing on targeted microbiological investigations, for example double-label isotopic experiments to simultaneously trace fungal biomass and growth kinetics [14], would provide an independent avenue for similar tests of the proposed model.

## Supporting Information

S1 File KML representation of fairy spiral sites. KML file viewable via Google Earth containing details of 20 sites across the continental United States exhibiting fairy spirals. Imagery captured through the USGS High Resolution Orthoimagery (HRO) program via Google Earth.
(KML)

S1 Table Characteristics of fairy spiral sites. Basic soil, drainage, and usage characteristics of the sites featured in S1 File.
(PDF)

S1 Text Numerical Methods and Classification Criterion. Detailed description of the numerical methods used to simulate the Gray-Scott system and the metrics used to classify simulation outcomes.
(PDF)
