# Supplementary material for: Spiral and Rotor Patterns Produced by Fairy Ring Fungi
Source: PLoS One. 2016 Mar 2;11(3):e0149254. doi: 10.1371/journal.pone.0149254 (PMC4774996; doi:10.1371/journal.pone.0149254)
Supplement: S1 Table — Basic soil, drainage, and usage characteristics of the sites featured in S1 File. (PDF) [file pone.0149254.s002.pdf]

Name	Latitude	Longitude	Soil Type	Soil drainage class	Usage
Spirals and rotors (MD, 2008)	39.525678	-77.768956	Lappans loam	Moderately well	Hay/pasture
Spiral and rotor (MD, 2008)	39.521958	-77.770853	Lappans loam	Moderately well	Hay/pasture
Rotors (MD, 2008)	39.658450	-77.938443	Murrill gravelly loam	Well	Playing field
Spiral (MD, 2008)	39.662446	-77.718208	Hagerstown-Duffield	Well	Lawn
Rotor (MD, 2008)	39.591227	-77.806804	Duffield silt loam	Well	Lawn
Isolated Spiral (MD, 2007)	38.245256	-76.484837	Beltsville silt loam	Moderately well	Playing field
Spirals and rotors (NE, 2015)	41.660943	-96.666218	Luton silt clay	Poorly	Playing field
Rotors (NE, 2015)	41.731352	-96.501243	Moody silty clay loam	Well	Lawn
Rotors (IN, 2005)	40.785628	-85.504061	Blount silt loam	Somewhat poorly	Playing field
Rotor (IN, 2005)	41.145908	-85.478408	Morley loam	Moderately well	Lawn
Curled tip (IN, 2012)	40.415808	-86.948939	Carmi loam	Well	Airport
Rotors (TN, 2009)	35.884468	-83.840581	Bloomingdale silt loam	Poorly	Hay/pasture
Weak rotors (TN, 2006)	35.883314	-83.809123	Swafford silt loam	Moderately well	Lawn
Weak rotors (TN, 2006)	35.857718	-83.844392	Swafford silt loam	Moderately well	Hay/pasture
Paired rotors (TN, 2011)	36.623685	-82.290367	Frederick silt loam	Well	Hay/pasture
Small spiral (IA, 2009)	41.674894	-93.700977	Dickinson	Somewhat excessively	Playing field
Spiral tip on tree (VA 2011)	37.586859	-79.755832	Wolfgap loam	Well	Hay/pasture
Spirals (VA, 2011)	37.598078	-79.568383	Wolfgap loam	Well	Hay/pasture
Weak rotors (MT, 2006)	46.838733	-114.041832	Moiese gravelly loam	Excessively	Golf course
Spiral (MT, 2006)	46.843757	-114.014977	Urban	Unrated	Playing field

Table 1: Basic soil and usage characteristics for the 20 fairy spiral and rotor sites documented in S1 KML.
