# Supplementary material for: Spiral and Rotor Patterns Produced by Fairy Ring Fungi
Source: PLoS One. 2016 Mar 2;11(3):e0149254. doi: 10.1371/journal.pone.0149254 (PMC4774996; doi:10.1371/journal.pone.0149254)
Supplement: S1 Text — Detailed description of the numerical methods used to simulate the Gray-Scott system and the metrics used to classify simulation outcomes. (PDF) [file pone.0149254.s003.pdf]

Numerical Methods and Classification Criteria

Numerical solutions to Equations (5)–(6) were generated for $N = 20,000$ total time steps of size $\Delta t = 0.1$ in a forward Euler solver using MATLAB's `del2` discretized approximation of the Laplacian. The simulation domain was taken to be a $[0, 2] \times [0, 2]$ Cartesian grid with 256 spatial sites per axis, in line with other numerical studies of the system [1, 2, 3]. Spot checks confirmed that these simulations were well resolved spatially and of sufficient duration to allow the system's dynamics to fully develop. Simulations were run using both Neumann (no flux) and periodic boundary conditions: the results were robust to the choice of boundary condition.

The line source was considered because spirals are known to form around the free end of linear fronts in similar systems [4, 5]. The center of rotation of spirals is not generally fixed, meaning that the dynamics can potentially wander off the finite simulation domain. To pin the center of the spiral and prevent such wandering, we artificially held resource concentrations to be zero in a disk of radius 0.01 centered at $(x, y) = (1, 1)$ [6, 7]. Fungal biomass was introduced at a high concentration (0.9, a high enough value to ensure the initial colony survived) in a narrow rectangular domain, $[0, 1] \times [1, 1.0156]$, with one vertex lying within the zero-resource region. Resources were set to zero just below the line of high biomass, namely in the region $[0, 1] \times [0.922, 1]$, so as to bias the rotation of the spiral clockwise. In all other regions, the initial resource concentration was set to its maximum value, and fungal biomass to zero. To prevent front-interaction from occurring, we only used no flux boundary conditions in these simulations.

The multiple-point initial conditions create a situation where the only mechanism for spiral formation lies in the interaction of propagating fronts. Fungal biomass was set to 0.9 in a circle of radius of 1×10^{-4} dimensionless length units centered on five randomly selected locations. The remainder of the domain was initialized with zero fungal biomass and maximum resource concentrations. Symmetry prevents spiral formation from occurring at the isolated innoculation sites, meaning that any observed spirals must form due to front-interaction. Spot checks throughout the simulations confirmed that these interactions occurred and generated spirals.

The limiting values of replenishment and mortality at which spirals were formed for both sets of initial conditions were determined through ad hoc numerical experimentation. These extrema provided bounds on the exploration of parameter space. Both g and m were linearly varied between these bounds, and time domain simulations run for each factorial combination of the parameters. Both parameters were treated as spatially homogeneous and temporally constant throughout the simulation domain.

The tested parameter combinations were classified as supporting spiral/rotor formation only if spirals/rotors were found in the long term biomass dynamics. Transient spiral dyanmics that lead to a steady-state static biomass pattern were considered negative results. Similarly, parameter combinations were only considered to support fairy rings formation if (i) isolated rings were sustained

and propagated in the long term, or (ii) multiple rings disappeared through mutual annihilation with other fronts. Note that we only mark the existence of spirals at a particular point in time. Analyzing the stability of spiral waves in isolation is difficult [8] and analyzing the stability of spiral waves that are interacting with other fungal fronts is not mathematically feasible. We therefore assume that if spirals and/or rotors have persisted until the end of a simulation, then these dynamics represent long-term, non-transient behaviors of the system. Cases in which rings propagated a short distance and then decayed to the ground state before the end of the simulation were treated as negative results.

Bibliography

[1] Davidson FA, Sleeman BD, Rayner ADM, Crawford JW, Ritz K. Context-Dependent Macroscopic Patterns in Growing and Interacting Mycelial Networks. Proceedings of the Royal Society B: Biological Sciences. 1996 Jul;263(1372):873–880.

[2] Davidson FA. Chaotic wakes and other wave-induced behavior in a system of reaction–diffusion equations. International Journal of Bifurcation and Chaos. 1998;8(6):1303–1313.

[3] Pearson J. Complex patterns in a simple system. Science. 1993;261:189–192.

[4] Fife PC. Understanding the patterns in the BZ reagent. Journal of Statistical Physics. 1985;39(5-6):687–703.

[5] Meron E, Pelcé P. Model for spiral wave formation in excitable media. Physical review letters. 1988;60(18):1880–1883.

[6] Ikeda T, Yashima M, Uchida T, Hough D, Fishbein MC, Mandel WJ, et al. Attachment of meandering reentrant wave fronts to anatomic obstacles in the atrium role of the obstacle size. Circulation Research. 1997;81(5):753–764.

[7] Shajahan TK, Sinha S, Pandit R. Spiral-wave dynamics depend sensitively on inhomogeneities in mathematical models of ventricular tissue. Physical Review E. 2007;75(1):011929.

[8] Barkley D. Linear stability analysis of rotating spiral waves in excitable media. Physical review letters. 1992;68(13):2090–2093.
